# Leishmaniasis Caused by *Leishmania major* on the Glans Penis: A Case Report

**Published:** 2019

**Authors:** Mahdi MOSAYEBI, Mehdi MOHEBALI, Aliasghar FARAZI, Mohammad Reza SHIRZADI, Davood AKHLAGHI, Reza HAJHOSSEIN, Samira ELIKAEE

**Affiliations:** 1. Department of Medical Parasitology and Mycology, School of Medicine, Arak University of Medical Sciences, Arak, Iran; 2. Health Center Laboratory, University of Medical Sciences, Arak, Iran; 3. Department of Medical Parasitology and Mycology, School of Public Health, Tehran University of Medical Sciences, Tehran, Iran; 4. Research Center for Endemic Parasites of Iran, Tehran University of Medical Sciences, Tehran, Iran; 5. Department of Infectious Diseases, School of Medicine, Arak University of Medical Sciences, Arak, Iran; 6. Communicable Diseases Management Center, Ministry of Health and Medical Education, Tehran, Iran

**Keywords:** Leishmaniasis, Glans penis, Genital, Iran

## Abstract

Cutaneous leishmaniasis (CL) is one of the prevalent parasitic diseases in Iran principally caused by two species, *Leishmania major* and *L. tropica*. Here, we present a rare case of a congenital form of CL around the glans penis from the central part of Iran in 2017. A 24-yr-old male patient from the central part of Iran presented with biennial ulceration of the glans penis. Diagnostic methods included physical and preclinical examination, microscopic observation, leishmanin skin test (LST), and serological tests including direct agglutination test (DAT). Nested PCR and sequencing analysis were used on the positive smears for confirmation of CL and *Leishmania* species identification. The preclinical results were normal, and no anti-*Leishmania* antibodies were detected in the peripheral blood of the patient using DAT. In abdominal ultrasonography, the spleen and liver size were normal. LST was positive (≥5 mm) after 72 h, and a few amastigote forms of *Leishmania* sp. were demonstrated under light microscopy. *L. major* was confirmed using nested PCR and sequencing analysis. The patient responded to oral administration of miltefosine (2.5 mg/kg/d) for 28 days. To the best of our knowledge, genital CL due to *L. major* has not been previously reported from Iran.

## Introduction

Various forms of leishmaniasis, a protozoan disease, caused by *Leishmania* species and in the life-cycle of parasite, sand flies (*Phlebotomus* and *Lutzomia*) have the role of transmitting disease to human and other hosts in many countries (1,2).

Cutaneous leishmaniasis (CL) in Iran is caused by two species of the *Leishmania* genus. *L. major* is present in many areas of Iran whereas *L. tropica* is present in some areas. Arak, the center of Markazi Province, is one of the areas where none of these species are common, but in several cities around the Markazi Province, such as Kashan, *L. major* is endemic. CL is often observed in the exposed body organs, and involvement of the glans penis has rarely been reported. Genital lesions of leishmaniasis are rare but have been described in several areas of the world (3).

In this paper, we present a case of chronic CL of the glans penis. This is the first report of glans penis leishmaniasis caused by *L. major* in Iran.

## Case report

A 24 yr old male electrician from Arak City, Markazi Province, the central part of Iran, presented with inflammatory crusty ulceration of the glans penis, in 2017. Before sample preparation, an informed consent was obtained from the patient. He was a resident of Arak City, where leishmaniasis is not common, but he had traveled to many places, for work, where leishmaniasis was prevalent. In those areas, he worked in half-built buildings and often slept and rested there.

**Fig. 1:**
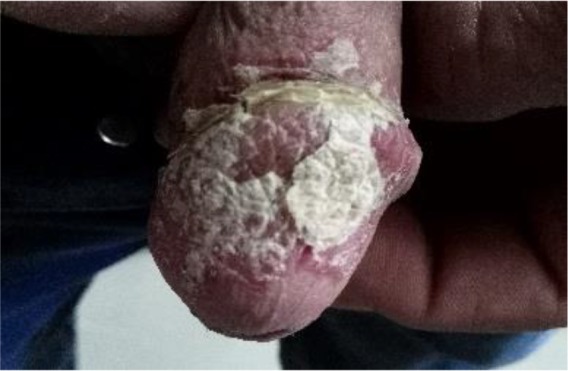
Ulcer of the glans penis in a patient who was referred to referral leishmaniasis lab. in 2017

Approximately two years before being referred to the authors, first, an ulcer occurred at the edge of the urethra, a flat spot without a clear bulge, and then spread to the right side of the glans. There had been no discharge or other genital symptoms. He was married before this time, but his wife divorced him after the illness continued. The patient was previously admitted to many health institutions, but the cause of the disease remained undiagnosed and was treated with nonspecific drugs and unsuccessful treatment. The patient was first referred to Arak Health Center Laboratory for the diagnosis of the causative fungal agent and candidal balanitis (Fig. 1). In addition to the sampling for the detection of fungal agents, samples were taken from the border of the ulcerated lesion to detect possible CL. Giemsa staining was done to detect amastigote forms of *Leishmania* parasites.

**Fig. 2:**
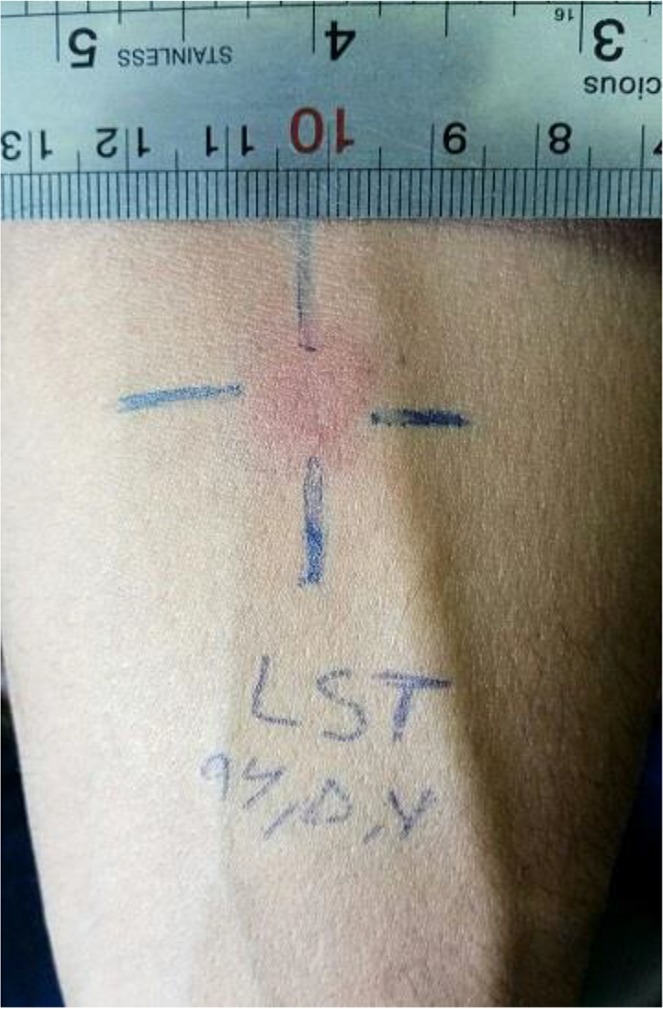
Positive leishmanin skin test (LST) with average induration 11 mm

**Fig. 3:**
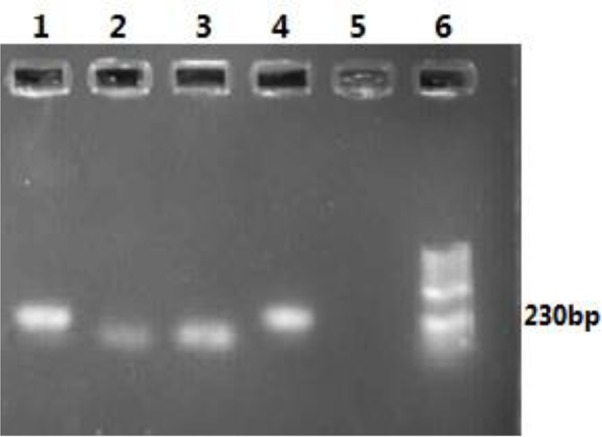
Nested PCR patterns of ITS2 gene obtained from the test sample and standard *Leishmania* stocks. Lane 1 is the sample of the patient; Lane 2: *Leishmania tropica* (MHOM/SU/74/K27); Lane 3: *Leishmania infantum* (MCAN/IR/07/Moheb-gh); Lane 4: *Leishmania major* (MRHO/IR/75/ER) as positive control; Lane 5: Negative control; Lane 6: 50bp size marker

The fungal examination was negative, but direct smears from the margin of the ulcer were positive, and Leishman bodies of *Leishmania* sp. were seen. Full body examination was unremarkable. In abdominal ultrasonography, all the organs were normal, and spleen size was 118 mm. No lymphadenopathy was observed in the cervical, supraclavicular, inguinal, and axillary sonography. The CBC, serology, blood biochemistry, hematology, and urinalysis parameters were within the normal range. In microbiology test, urine culture was negative. HIV was absent and no anti-*Leishmania* antibodies were detected in the serum of the patient using DAT, but leishmanin skin test (LST) was positive with an induration of approximately 11 mm (Fig. 2). The molecular detection of the parasites in the smear samples was done by nested PCR using the external primers, Leish out F (5′-AAA CTC CTC TCT GGT GCT TGC-3′) and Leish out R (5′-AAA CAA AGG TTG TCG GGG G-3′), and internal primers, Leish in F (5′-AAT TCA ACT TCG CGT TGG CC-3′) and Leish in R (5′-CCT CTC TTT TTT CTC TGT GC-3′), to amplify the final 230-bp fragment of the ITS2 gene (4). The ulcer was infected with *L. major* parasites (Fig. 3). For confirmation, the second amplified PCR product was sent to Bioneer Company (South Korea) for sequencing using a 3730XL Applied Biosystems. The sequence obtained has been submitted to Gen-Bank with accession number “MF614960.”

Since miltefosine has been used for *L. major* infections (5), the patient was orally administered capsules of miltefosine at the daily dose of 3 × 50 mg for 28 d; no side effects of miltefosine were seen, and the lesion healed within a month after the end of therapy. Only an erythematous spot remained at the ulcer site after recovery. No Leishman body was found on re-sampling.

## Discussion

Leishmaniasis presents with various clinical forms in the world (6). The presence of ulcer over the genital organs usually suggests sexually transmitted infections (3), but there are some diseases, infective or noninfective, that can cause an ulcer on the glans penis, one of them being leishmaniasis, though very uncommon. Investigation of any atypical lesion, especially chronic forms, is essential for CL, in an endemic area (7). There are many reports on the unusual presentations of CL worldwide and in Iran (8, 9). “Generally, genital lesions caused by leishmaniasis are rare but have been described in South America among miners and farmers (10).” There is also a report of vulval involvement (3). Reports of glans penis involvement, due to the low probability of sand fly feeding on that position, are rare. This is the first report of glans penis leishmaniasis caused by *L. major* in Iran. A case report from Venezuela presented two cases of CL in the genital area of men. In the two presented cases, the genital area localization claimed for accurate differential diagnosis (10). In one case, the species was not identified but was most likely to be *L. braziliensis* or *L. Mexicana*, both species being the major causes of CL in Venezuela (11, 12). In the other case, the causative agent of leishmaniasis on glans penis of the patient was *L. (V.) braziliensis* (13). From Turkey, a case of chronic hyperkeratotic lesion on penis has been reported. Microscopic test was positive for leishmaniasis but culture was negative. CL was performed by serologic assessment, but the species of *Leishmania* was not examined (14). In another case from Turkey, a 5 yr old boy with lesion on the glans penis was reported. The microscopic diagnosis was conducted by demonstrating the presence of Leishman-bodies in the smears and leishmaniasis was confirmed by biopsy and culture. Treatment was done with meglumine antimoniate injections (15). Moreover, in this report, the type of *Leishmania* species was not investigated.

The reported cases have been treated with different medications, and the patient treated by us was orally administered capsules of miltefosine at the daily dose of 3 × 50 mg for 28 d. Recently, some cases of leishmaniasis were successfully treated with miltefosine. “Miltefosine is a new oral anti-leishmanial drug that interacts with *Leishmania* phospholipid synthesis” (7).

## Conclusion

Briefly, in countries where this disease is prevalent, it is essential for the diagnosis of atypical ulcer properly, especially in the genital organs, the focus should be on the job, traveling history and the origin of the patient, comparing them to the life cycle and epidemiology of vectors and prevalent *Leishmania* species. For the definitive and final diagnosis, demonstration of the parasite by microscopy, culture, and/or molecular analysis by nested PCR and species identification by sequencing is needed. These were done in the present study.
